# Supplementary material for: Drosophila RhoGAP18B regulates actin cytoskeleton during border cell migration
Source: PLoS One. 2023 Jan 20;18(1):e0280652. doi: 10.1371/journal.pone.0280652 (PMC9858088; doi:10.1371/journal.pone.0280652)
Supplement: S1 Fig — The full-length PA coding sequence, with two EcoRI sites added in the 5’ and 3’ ends, was synthesized and subcloned to the pUAS-attB plasmid with EcoRI site. The DNA sequence synthesized for generating UAS-PA clone is: gaattcATGGCCGGCGATACGGAGAACAAAAGGGGCTATCGCACCATATTTCGCAGCATATCCCAGGTGTTCTACGCCAATGCAAAAAACTCGAGTAATACGAACAGCAGCAGCAGTAACAACAACAATAATATCATCAACTACAACAACAACACCATCGAGAACAACAACGAGGGAATCGGCCAACTAACAGTTACGCTCACCAGCATAACACCACCCACAGCCACATGGACGTCGACATCGCCCACAATCTCCACGTGCACAACTGCGTCCGCATCGGCGGGATCATCCACCGCATCCTTGTCGCCACTGGCTAGCAGATCAGCGCGATCGGCGGAGAATATATCCTGCAGCGAGTGTGGAAACCCAGATCCGTATCATGGCCAGGACATGGAGTTGGCAAAAGGTCAAGCCCTGACTCCCGAAGTGCCAGCAGTGCCGGTTCCAGCTGCCAGTGCGGATCAAAACCAGAAGCTAAGGGCCAGTTCCATGCTGGATCTGACCTCCAACCATCGCCATCAGAACGGCTTGAGGATTGTGCCAGTGACTGACATTCTGCGGGCGCAGCAGATCCAGGAGGACAATGAGGTGCAGCAGACGCAGCAGTCAACGATTCTCCAGCGCTATAAGAGCGTCTCCATTGGCAACCTGCTCGATCTGCCCGGCGAGGATGACAAGAAGTCCATCAAGAATCGCATGCGAATGAAGTTCGCCGTCAATAGCAATGTGTTTCGCATCAACCGTTCGCCGAAGAGCGAGAAGAAGTCGCGTTCACGTCGAGGCCAGGTGAACAGTTTGTACCTGGACGAGCAGAAGTATCCGCTCTTTGCCGCTCCGCTCAGCGCACTGGAGCTCAACATGACCGATCATCCGAATGTTCCACGTTTCGTGGTCGACGTTTGCGCCTACATCGAACAGCCGGAGTGCATCGAGCAGGACGGACTGTACCGCGCATCGGGCAACAAGGTTCTGGTGGACGAGCTGCGCAAGAAGCTGACGCACGTGTACGATCCCCGCTGGCTGAAGACGGACGATATCCACACGCTGACCAGTCTGCACAAGCAGTTCTTCCGAGAGCTCACCTCTCCGCTCATCACCCAGGAGGCCTACGAGCGACTGGGCAGGAGCCTTAACGACGACGCCGCCATCGAGCGGATGAGTTTGGCCTTTGACGACATGCCGGAGCCGAACCGCTCCACGCTGCGCTTCCTCATTAGGCACTTGAC CAGAGTTGCC GCTGCCAGCG CTTCGAATCCATGCCGTCCACCAATTTGG CCATCGTTTG GGGTCCTTGT CTGCTGAGTG CCAATCAGAT ACAGCTGGACATTGGACGCA TGAACATGCT GGCCAAGGTGCTGATCGAGA ACTATGATCG CATCTTTCATCCGGACAATG AGCGTCTAGT TTGTTAGgaattc. (DOCX) [file pone.0280652.s001.docx]

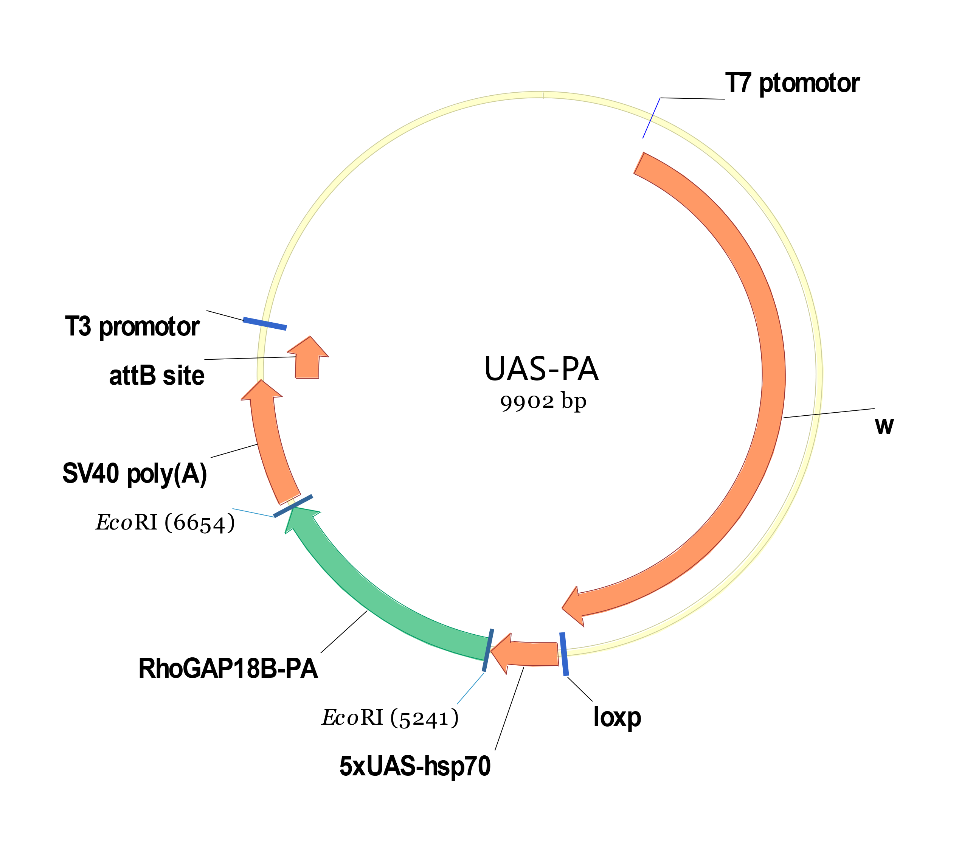


**S1 Fig.** The schematic diagram of UAS-PA plasmid. The full-length PA coding sequence, with two EcoRI sites added in the 5’ and 3’ ends, was synthesized and subcloned to the pUAS-attB plasmid with EcoRI site.

The DNA sequence synthesized for generating UAS-PA clone is: gaattcATGGCCGGCGATACGGAGAACAAAAGGGGCTATCGCACCATATTTCGCAGCATATCCCAGGTGTTCTACGCCAATGCAAAAAACTCGAGTAATACGAACAGCAGCAGCAGTAACAACAACAATAATATCATCAACTACAACAACAACACCATCGAGAACAACAACGAGGGAATCGGCCAACTAACAGTTACGCTCACCAGCATAACACCACCCACAGCCACATGGACGTCGACATCGCCCACAATCTCCACGTGCACAACTGCGTCCGCATCGGCGGGATCATCCACCGCATCCTTGTCGCCACTGGCTAGCAGATCAGCGCGATCGGCGGAGAATATATCCTGCAGCGAGTGTGGAAACCCAGATCCGTATCATGGCCAGGACATGGAGTTGGCAAAAGGTCAAGCCCTGACTCCCGAAGTGCCAGCAGTGCCGGTTCCAGCTGCCAGTGCGGATCAAAACCAGAAGCTAAGGGCCAGTTCCATGCTGGATCTGACCTCCAACCATCGCCATCAGAACGGCTTGAGGATTGTGCCAGTGACTGACATTCTGCGGGCGCAGCAGATCCAGGAGGACAATGAGGTGCAGCAGACGCAGCAGTCAACGATTCTCCAGCGCTATAAGAGCGTCTCCATTGGCAACCTGCTCGATCTGCCCGGCGAGGATGACAAGAAGTCCATCAAGAATCGCATGCGAATGAAGTTCGCCGTCAATAGCAATGTGTTTCGCATCAACCGTTCGCCGAAGAGCGAGAAGAAGTCGCGTTCACGTCGAGGCCAGGTGAACAGTTTGTACCTGGACGAGCAGAAGTATCCGCTCTTTGCCGCTCCGCTCAGCGCACTGGAGCTCAACATGACCGATCATCCGAATGTTCCACGTTTCGTGGTCGACGTTTGCGCCTACATCGAACAGCCGGAGTGCATCGAGCAGGACGGACTGTACCGCGCATCGGGCAACAAGGTTCTGGTGGACGAGCTGCGCAAGAAGCTGACGCACGTGTACGATCCCCGCTGGCTGAAGACGGACGATATCCACACGCTGACCAGTCTGCACAAGCAGTTCTTCCGAGAGCTCACCTCTCCGCTCATCACCCAGGAGGCCTACGAGCGACTGGGCAGGAGCCTTAACGACGACGCCGCCATCGAGCGGATGAGTTTGGCCTTTGACGACATGCCGGAGCCGAACCGCTCCACGCTGCGCTTCCTCATTAGGCACTTGAC CAGAGTTGCC GCTGCCAGCG CTTCGAATCCATGCCGTCC
ACCAATTTGG CCATCGTTTG GGGTCCTTGT CTGCTGAGTG CCAATCAGAT ACAGCTGGACATTGGACGCA TGAACATGCT GGCCAAGGTGCTGATCGAGA ACTATGATCG CATCTTTCATCCGGACAATG AGCGTCTAGT TTGTTAGgaattc
