# Supplementary material for: Drosophila RhoGAP18B regulates actin cytoskeleton during border cell migration
Source: PLoS One. 2023 Jan 20;18(1):e0280652. doi: 10.1371/journal.pone.0280652 (PMC9858088; doi:10.1371/journal.pone.0280652)
Supplement: S2 Fig — (A-G) Stage 10 egg chambers of labeled genotypes show the c306-Gal4 expression patterns by GFP (Green). The arrows show the positions of the border cell clusters. (H) Quantitation of border cell migration. The x-axis denotes the percentage of stage 10 egg chambers for each genotype with each degree of migration. The extent of migration for all stage-10 egg chambers examined is measured as 0–5% (blue, no migration), 6–25% (yellow), 26–50% (pink), 51–75% (green), 76–100% (black, complete migration). The number of egg chambers examined for each genotype is given (n). Expression of PA RNAi driven by c306-Gal4 disrupts border cell migration, but expression of PB or PC/PD RNAi did not affect border cell migration. Expression of PA also disrupts border cell migration and can rescue the migration defect caused by PA RNAi expression. (DOCX) [file pone.0280652.s002.docx]

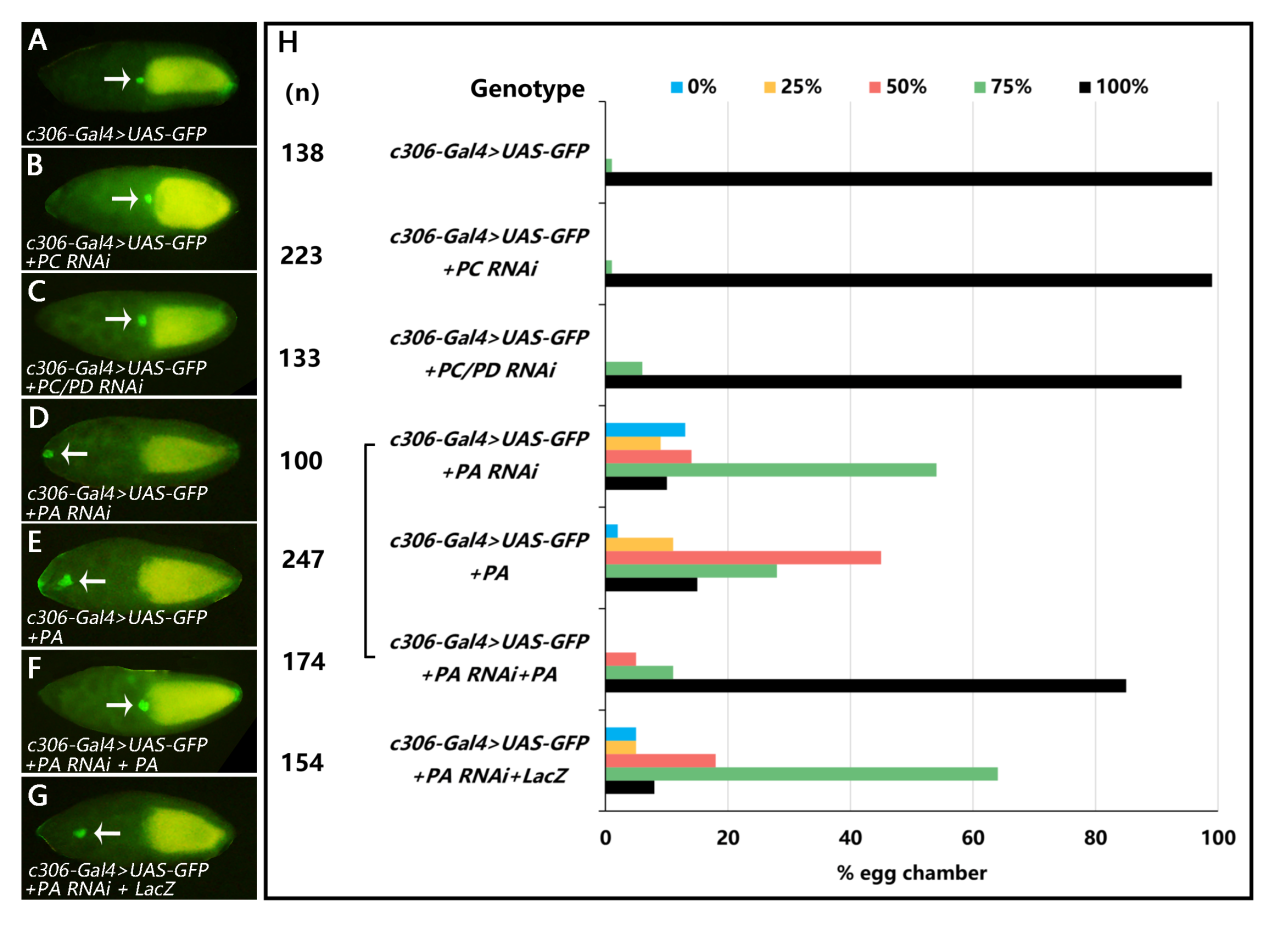


**S2 Fig.** Border cell migration degrees caused by RNAi or transgene expressions with *c306-Gal4*. (A-G) Stage 10 egg chambers of labeled genotypes show the *c306-Gal4* expression patterns by GFP (Green) . The arrows show the positions of the border cell clusters. (H) Quantitation of border cell migration. The x-axis denotes the percentage of stage 10 egg chambers for each genotype with each degree of migration. The extent of migration for all stage-10 egg chambers examined is measured as 0-5% (blue, no migration), 6-25% (yellow), 26-50% (pink), 51-75% (green), 76-100% (black, complete migration). The number of egg chambers examined for each genotype is given (n). Expression of PA RNAi driven by *c306-Gal4* disrupts border cell migration, but expression of PB or PC/PD RNAi did not affect border cell migration. Expression of PA also disrupts border cell migration and can rescue the migration defect caused by PA RNAi expression.
