# Supplementary material for: Drosophila RhoGAP18B regulates actin cytoskeleton during border cell migration
Source: PLoS One. 2023 Jan 20;18(1):e0280652. doi: 10.1371/journal.pone.0280652 (PMC9858088; doi:10.1371/journal.pone.0280652)
Supplement: Attachment — Submitted filename: Response to Reviewers.docx [file pone.0280652.s004.docx]

Reviewer #1: The paper has improved in revision and is now clearer. Here are two minor corrections on standard English language usage in response to question 5: section 3.4 heading should say "genetically interacts" instead of "genetic interact" figure 4 legend should say "defects in RhoGAP18B-PA knockdown in border cells" instead of "defects of PA knckdown border cells"

We have corrected the two language errors in the revised manuscript. Thank you for your comments.

Reviewer #2: All my comments have been addressed. And recommend for the acceptance of this manuscript for publication.

Thank you for your comments.
